# Natural Forest Biomass Estimation Based on Plantation Information Using PALSAR Data

**DOI:** 10.1371/journal.pone.0086121

**Published:** 2014-01-21

**Authors:** Ram Avtar, Rikie Suzuki, Haruo Sawada

**Affiliations:** 1 Institute of Industrial Science, The University of Tokyo, Tokyo, Japan; 2 Research Institute for Global Change, Japan Agency for Marine-Earth Science and Technology, Yokohama, Japan; 3 United Nations University Institute for Sustainability and Peace, Tokyo, Japan; University of Missouri, United States of America

## Abstract

Forests play a vital role in terrestrial carbon cycling; therefore, monitoring forest biomass at local to global scales has become a challenging issue in the context of climate change. In this study, we investigated the backscattering properties of Advanced Land Observing Satellite (ALOS) Phased Array L-band Synthetic Aperture Radar (PALSAR) data in cashew and rubber plantation areas of Cambodia. The PALSAR backscattering coefficient (σ^0^) had different responses in the two plantation types because of differences in biophysical parameters. The PALSAR σ^0^ showed a higher correlation with field-based measurements and lower saturation in cashew plants compared with rubber plants. Multiple linear regression (MLR) models based on field-based biomass of cashew (C-MLR) and rubber (R-MLR) plants with PALSAR σ^0^ were created. These MLR models were used to estimate natural forest biomass in Cambodia. The cashew plant-based MLR model (C-MLR) produced better results than the rubber plant-based MLR model (R-MLR). The C-MLR-estimated natural forest biomass was validated using forest inventory data for natural forests in Cambodia. The validation results showed a strong correlation (R^2^ = 0.64) between C-MLR-estimated natural forest biomass and field-based biomass, with RMSE  = 23.2 Mg/ha in deciduous forests. In high-biomass regions, such as dense evergreen forests, this model had a weaker correlation because of the high biomass and the multiple-story tree structure of evergreen forests, which caused saturation of the PALSAR signal.

## Introduction

The global demand for forests and forest products is increasing because of population growth. To meet this demand, the importance of plantations has increased because they have high wood-production rates compared with natural forests [1]. Plantation forest production meets two-thirds of the global industrial roundwood demand [2]. Therefore, plantation forests are reducing the pressure on natural forests for wood and other resources, but they simultaneously pose a threat by replacing natural forests [3]. The objective of plantation forests is to produce timber and other wood products and to generate greater financial returns because of faster growth than occurs in natural forest regeneration [4]. Plantations can also be used for land rehabilitation, soil conservation, carbon sequestration, and water conservation and they can provide socio-economic benefits, minimising the poverty of local people in developing countries [5]. Different types of plantations are established for different purposes, and most are planted as monocultures [6]. These monoculture plantations, such as rubber, teak, eucalyptus, cashew, oilpalm, acacia etc., have an adverse impact on biodiversity and ecosystem services [7]; [8]. A recent report by Forest Resource Assessment [2] showed that plantation forests account for 7% (264 million ha) of total global forest area. During the period from 2005 to 2010, the area of planted forests increased by about 5 million ha per year worldwide. In Asia, plantation area has increased rapidly in recent years [2]. Plantations have become an interesting topic of research under the current scenario of climate change. Carbon (C) sequestration by plantation species has become an essential concern because consideration of its effects and calculation of C-credits during climate change negotiations require estimates of C sequestration by plantation species. Therefore, examinations of plantation biophysical parameters are necessary to precisely calculate biomass. To improve sustainability, a certain ratio between plantation and natural forests is necessary. Cashew and rubber plants are the major plantation species in Cambodia [9]. Both species are exotics, and the two types of plantations differ in their canopy cover, tree size, orientation of branches, and tree distribution.

Tropical forests contain about 40% of the carbon found in terrestrial biomass [10]; [11]; [12]. The most alarming global threats in the forest sector are deforestation and forest degradation, which contribute approximately 20% of global anthropogenic CO_2_ emissions [13]. Curbing deforestation through improved forest management practices could offer one of the most cost-effective methods of emission reduction [14] and could be achieved by implementing Reducing Emissions from Deforestation and forest Degradation Plus (REDD+) policies. REDD+ schemes can help to reduce emissions of CO_2_ by avoiding deforestation and could also protect biodiversity and forest-derived ecosystem services. These schemes can also improve the socio-economic conditions of local and indigenous people who depend on forests for their livelihood [15]. Observations of forests and their biomass distributions are the most important requirement for REDD+ [16]; [17]; [18]; [19]. Implementation of REDD+ policies will provide financial incentives to signatory countries that conserve and enhance their forest biomass. Because of this, precise and reliable methods are needed to estimate forest carbon stocks at national level to assess the economic benefits [20]; [21]. Forest carbon pools consist of trunks, branches, leaves, litter, dead wood, roots and soil carbon. However, most studies have focused on above ground biomass (AGB) because this is relatively large pool [16]. In tropical forests worldwide, about 50% of the total carbon is stored in aboveground biomass and 50% is stored in the top 1 m of the soil [22]. Satellite-based remote-sensing techniques have played a crucial role in biomass monitoring; they are more cost and time efficient than collecting forest inventory data and can provide spatial and temporal information on forest biomass distribution [23]; [24].

Previous studies have demonstrated that remote-sensing techniques can be used for biomass estimation using optical [25]; [26]; [27], Synthetic Aperture Radar (SAR) [28]; [29]; [30]; [31], and Light Detection And Ranging (LiDAR) data [32]; [33]; [34]; [35]; [36]; [37]. However, these techniques have several limitations; optical data saturate in low-biomass regions [38]; [39]; [40] and are not effective in tropical regions because of frequent cloud coverage [41]; [42]. SAR data are limited by terrain, speckle, and saturation [28]; [30]; [31]. LiDAR data are very expensive and cannot provide global coverage with plant species information [20]; [43]. In tropical countries, SAR is the most effective technique because it can penetrate clouds and acquire data throughout the year irrespective of cloud coverage and shadows [41].

Previous studies have shown that SAR data are dependent on various properties of the radar system (wavelength, polarisation, and incidence angle) and of the target (soil moisture, surface roughness, vegetation structural properties, vegetation water content, and geometric properties) [44]; [45]; [46]. Forest structure and the distribution of biomass throughout the forest canopy have significant effects on radar backscatter (σ^0^), which depends on the size distribution, orientation, and density of the scatterers [47]. A number of studies have been undertaken to understand the behaviour of multi-frequency (X-band, C-band, L-band, and P-band) and multi-polarisation (HH, HV, VH, and VV) SAR data with respect to forest biophysical parameters [48]; [49]; [50]; [51]; [52]. Longer wavelengths have proven more effective for biomass estimation. This study demonstrates the capability of Phased Array L-band Synthetic Aperture Radar (PALSAR) dual polarimetric (HH and HV) data for forest biomass estimation because of the high penetration of L-band SAR. The usefulness of PALSAR polarimetric data for discriminating different types of scattering from targets has been noted in previous studies [46]; [53]; [54]; [55].

Various studies have attempted to quantify above-ground biomass (AGB) at local to global scales [21]; [46]; [54]; [56]; [57]. However, to date, no study has used plantation information in the estimation of biomass in natural forests. In this study, we developed an empirical model based on plantation biomass and PALSAR σ^0^ and applied it to natural forests in Cambodia. The primary aim of this research was to compare the biophysical parameters of cashew and rubber plants and to determine the relationship between PALSAR σ^0^ and field-measured AGB. The second aim was to develop multiple linear regression (MLR) models based on a combination of plantation types (cashew, rubber, and mixed) and to compare the sensitivity of plantation-based natural forest AGB estimates using PALSAR data in Cambodia.

### Study area

Both cashew and rubber plantation areas are situated in Kampong Cham Province of Cambodia. It is about 90 km from Phnom Penh and lies between 12°2′17″N to 12°6′7″N latitude and 104°59′51″E to 105°4′23″E longitude (cashew plantation area) and 12°5′36″N to 12°8′20″N latitude and 105°41′9″E to 105°44′11″E longitude (rubber plantation area). Cambodia is a tropical country with the rainy season from May to October and dry season from November to April. The minimum and maximum temperatures of the study areas are about 21°C and 35°C, respectively. The mean annual rainfall ranges from 1500 to 1800 mm [58].

**Figure 1:**
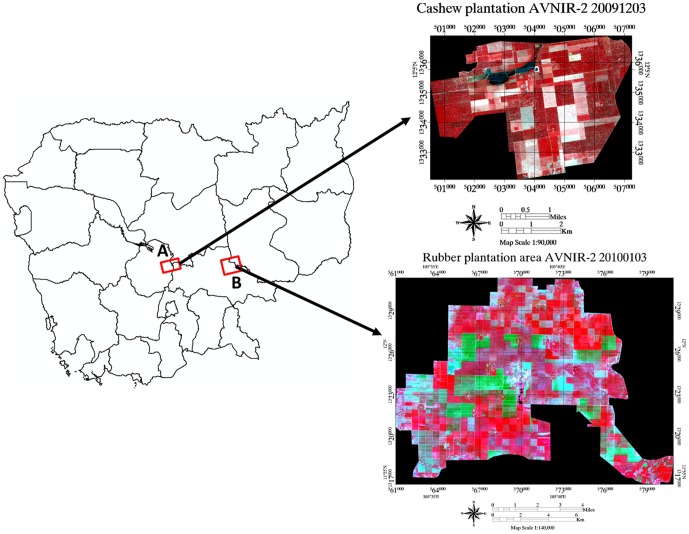
Location of the study area (A: Cashew plantation area, B: Rubber plantation area) AVNIR-2 data false color composite (Red:NIR, G:Red, B = Green color composite).

We studied the cashew and rubber plantation area managed by Agrostar private company and Chup rubber Plantation Company of Kampong Cham province respectively (Figure 1). Cashew (*Anacardium occidentale*) is an evergreen fast growing tropical tree that grows up to 10–15 m height with a diameter at breast height (DBH) of 100 cm under favorable growing conditions [59]. Cashew plants show three stages of their growth as follows: juvenile (1–3 year), young (4–8) and mature/old (9–20). Rubber (*Hevea brasilliensis*) is a fast growing exotic species in Cambodia that grows up to 20–25 m height with a 50–60 cm DBH. Rubber is the second largest source of agricultural export after rice in Cambodia [60]. Cambodia has produced 42000 M ton of dry rubber in 2010 [13]. Rubber plant can be tapped approximately 4 to 6 years after planting. Cambodia has ideal conditions for the commercial cultivation of rubber and cashew plants [61]. Cashew and rubber plantation area are on flat areas and homogeneous spreads (same age group and same inter tree spacing) about 2,000 ha and 16000 ha respectively. This lack of topographic effects makes PALSAR more applicable in the measurement of plants biophysical parameters [42].

## Methodology

### a. Satellite data used

Analysis of satellite archive data is useful for forest inventory data collection because it can provide primary information about the starting of plantation year, extent of change in the plots and various conditions of forests [62]. Landsat archive data were downloaded from Glovis (www.glovis.usgs.gov). Three scenes of PALSAR fine beam dual (FBD) 1.5 level data acquired on December 5^th^, 2010 (cashew plantation area), November 18^th^, 2010 (rubber plantation area) and November 1^st^, 2010 (natural forest area) with 34.3° off-nadir angle were used in this study [63]. PALSAR data in dry season was selected based on finding from Lucas et al. (2010) [46] to minimize effects of soil moisture on backscattering properties. Landsat archive data from 1990 to 2010 were used to establish sampling plots using visual image interpretation. Landsat archive data also provide information about the trend of cashew and rubber plantation activity and history of the starting of cashew and rubber plantation. Landsat archive shows that most of the plantation was started in between 1995 and 2000, and it was confirmed by interviewing local workers. Therefore, archive data shows the importance of multi-temporal satellite data to know the historical information about the land use/cover pattern and their changes. Advanced Visible and Near Infrared Radiometer Type-2 (AVNIR-2) data with 10 m resolution acquired in 2010 were also used to identify the spectral signature of plantation area based on Normalized Difference Vegetation Index (NDVI) value.

### b. Field data collection

**Figure 2:**
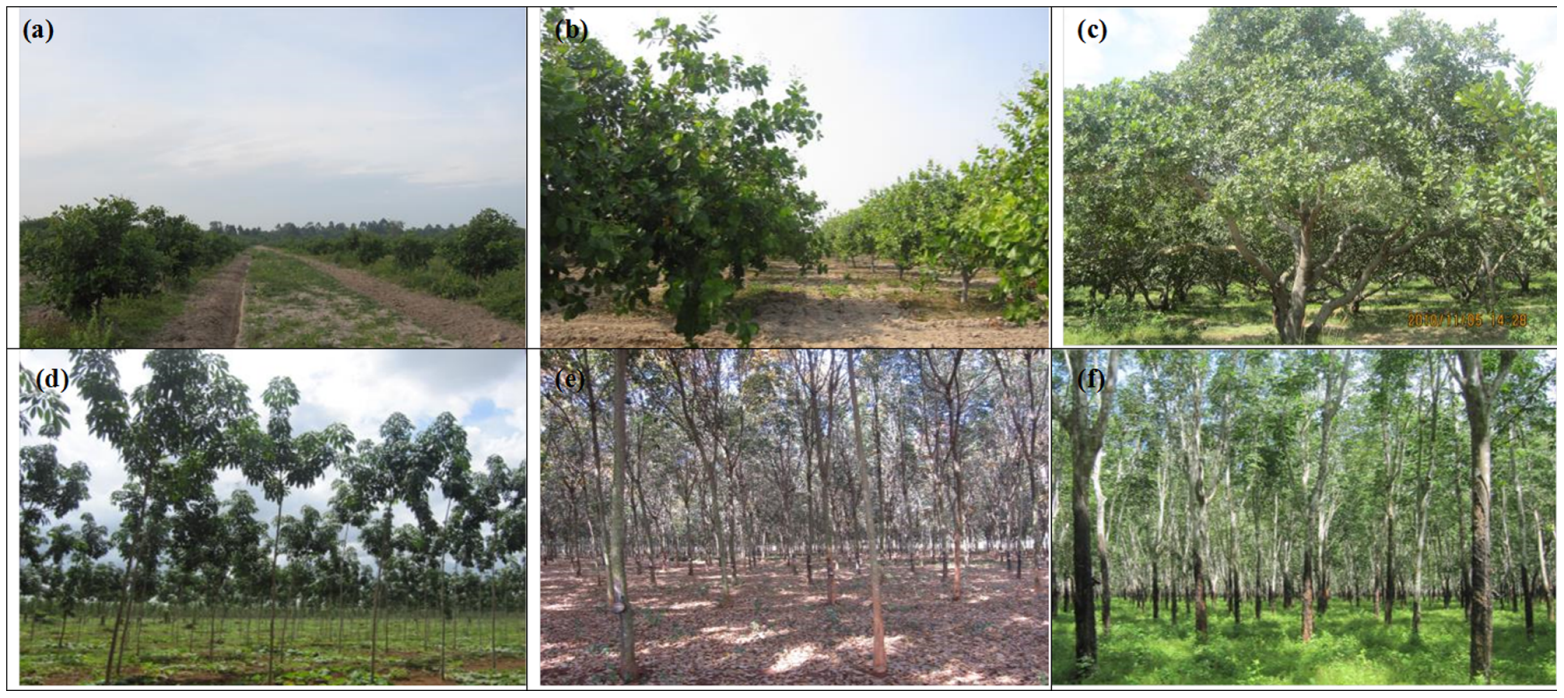
Cashew plantation area with different growth stages (a) juvenile, (b) young, (c) old and rubber plantation area with (d) juvenile, (e) young, (f) old growth stages.

Forest inventory data is useful for forest management planning and to identify the management schedules of tree stands for sustainable thinning [64]. Fieldwork was conducted in November, 2010 with the help of Forestry Administration (FA) of Cambodia, which is a government authority under the Ministry of Agriculture Forestry and Fisheries (MAFF). We also took permission from Chup Rubber Plantation Company to conduct the field survey. This field study did not involve any endangered species and no vertebrate study was conducted. A total of twenty one and nineteen sampling plots were collected to measure and estimate specific biophysical parameters of cashew and rubber plants respectively. A stratified random sampling procedure was applied to ensure that the sampling measurements captured all possible age classes of cashew and rubber plants across the plantation area. In each sampling plot tree density, DBH, height, plantation year, and crown diameter were measured. The four corners of the plots were established in the field using Garmin Global Positioning System (GPS) 60CSx, with plot size 30×30 m to access the accuracy of PALSAR 12.5×12.5 m pixel. Eighteen sampling plots data were also collected from natural forest area during January 2011 for validation. It means, 18 sampling plots data were used to validate the natural forests biomass predicted using cashew plants based MLR model (C-MLR) and rubber plants based MLR model (R-MLR). GPS locations and photos of the plots were also collected during the field visit. Figure 2 shows the snapshot picture of the cashew and rubber plantation area with different growth stages i.e. juvenile, young and mature. Cashew plantation area (Figure 2c) shows regular spacing between two plants with multiple branches from the ground level. Rubber plantation area (Figure 2f) also shows regular spacing between two plants with high tree density. Table 1 shows the comparison between the characteristics of natural forest and plantation forest. Collection of forest inventory data in plantation area is more convenient with less uncertainty compared to natural forest (based on field data collection).

**Table 1:** Comparison between natural and plantation forest characteristics.

	Natural Forests	Plantation Forest
1.	Multi-story (Complex structure)	Single-story (Simple structure)
2.	Multi species (Flora and fauna)	Single species (mono-culture)
3.	High biomass density (sequester more carbon)	Less biomass density (sequester less carbon)
4.	Continuous plant growth	Initial plant growth very fast
5.	Forest inventory data collection time, cost and labour intensive	Forest inventory data collection time and cost efficient
6.	Inventory data has high uncertainties	Inventory data has fewer uncertainties

The biomass of cashew plants were calculated using the following allometric equation (equation 1) [59].


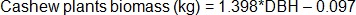
(1)

The biomass of rubber plants were calculated using the following allometric equation (equation 2) [65].


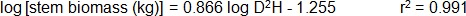
(2)


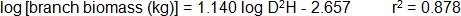
(3)


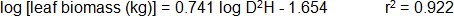
(4)

where, *H* and *D* represent *height* and *DBH* of rubber plant respectively. The total AGB for a tree was the sum of the stem biomass, branch biomass and leaves biomass.

The biomass of natural forest was calculated using the following allometric equation (equation 5–7) [66].


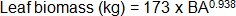
(5)


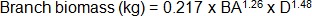
(6)


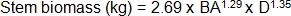
(7)

where, *BA* and *D* represents basal area (m^2^) and DBH of the tree respectively. The total AGB for a tree was the summation of the stem biomass, branch biomass and leaves biomass.

### c. Satellite data processing

AVNIR-2 data and Landsat data were used for selection of sampling sites. PALSAR FBD data were processed for converting digital number (DN) to σ^0^. The backscattering coefficient was calculated using the following equation [67].


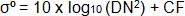
(8)

Where, CF is the calibration factor and its value for PALSAR FBD 1.5 L data is -83 because it was acquired after 9^th^ January, 2009. The PALSAR data were co-registered with AVNIR-2 data based on field collected ground control points (GCPs). Later the data were frost filtered with window size of 5×5 to reduce speckle noise [68]. We have considered the climatic conditions of the area during the analysis of PALSAR data to minimize climatic effects.

### d. Statistical Analysis

**Figure 3:**
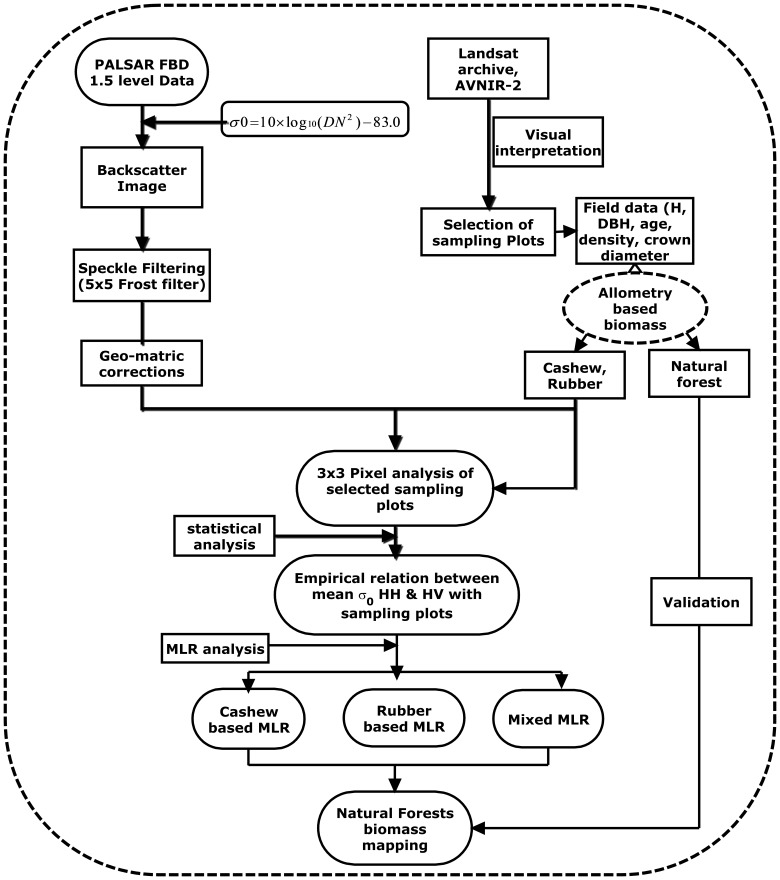
Flow chart of the methedology.

The mean σ^0^ was calculated for each sampling plots with 3×3 pixels size. We used 3×3 pixels window size to minimize the positional inaccuracies of GPS and geo-coding. The relationships between σ^0^ and biophysical parameters were analyzed using logarithmic equation because of the backscattering value of PALSAR is in logarithmic scale. Correlation analysis and Multi-linear regression (MLR) model approach have been applied. Field based biophysical parameters and PALSAR backscattering in HH, HV mode were used in MLR approach. MLR approach has also been used by previous researcher and good results have been achieved in their studies [46]; [47]; [50]; [51]; [54]; [65]; [69]; [70]. MLR analysis using the stepwise forward method was conducted relating the σ^0^ of PALSAR to corresponding field calculated biomass [67]; [71]. We have generated three MLR models using cashew (C-MLR), rubber (R-MLR) and mixed (M-MLR) plantation types. Here mixed means joining of the plots from pure cashew and pure rubber plantations. These models have been applied to the PALSAR data to estimate biomass of natural forests. Finally validation was used to evaluate the accuracy of the model by comparing PALSAR estimated AGB to the field derived AGB. Figure 3 illustrates the flow chart of methodology adopted in this study.

## Results and Discussion

### a. Comparison of cashew and rubber plant biophysical parameters

A correlation matrix among the various biophysical parameters of cashew and rubber plants is summarised in Tables 2 and 3. The correlation matrix shows that all respective biophysical parameters are well correlated except tree density. Basic information on planted clones, area of plantation, and age of trees was obtained from plantation owners and workers with the help of an interpreter. Information about the plantation history and agro-forestry practices was also collected [72]; [73].

**Table 2:** Pearson's correlation matrix of various biophysical parameters of cashew plants.

	Age (years)	Crown Dia (m)	Tree Density	Height (m)	DBH at 10 cm (cm)	DBH at 1.3 m (cm)	Biomass (Mg/ha)
**Age (years)**	1						
**Crown Dia (m)**	0.92	1					
**Tree Density**	−0.58	−0.64	1				
**Height (m)**	0.91	0.90	−0.65	1			
**DBH at 10 cm (cm)**	0.94	0.93	−0.64	0.93	1		
**DBH at 1.3 m (cm)**	0.96	0.94	−0.66	0.89	0.96	1	
**Biomass (Mg/ha)**	0.97	0.91	−0.53	0.88	0.95	0.97	1

**Table 3:** Pearson's correlation matrix of various biophysical parameters of rubber plants.

	Age (years)	Crown Dia. (m)	Tree density	Height (m)	DBH (cm)	Biomass (Mg/ha)
**Age (years)**	1					
**Crown Dia. (m)**	0.70	1				
**Tree density**	−0.48	−0.24	1			
**Height (m)**	0.92	0.79	−0.45	1		
**DBH (cm)**	0.91	0.87	−0.40	0.96	1	
**Biomass (Mg/ha)**	0.95	0.67	−0.35	0.95	0.92	1

**Figure 4:**
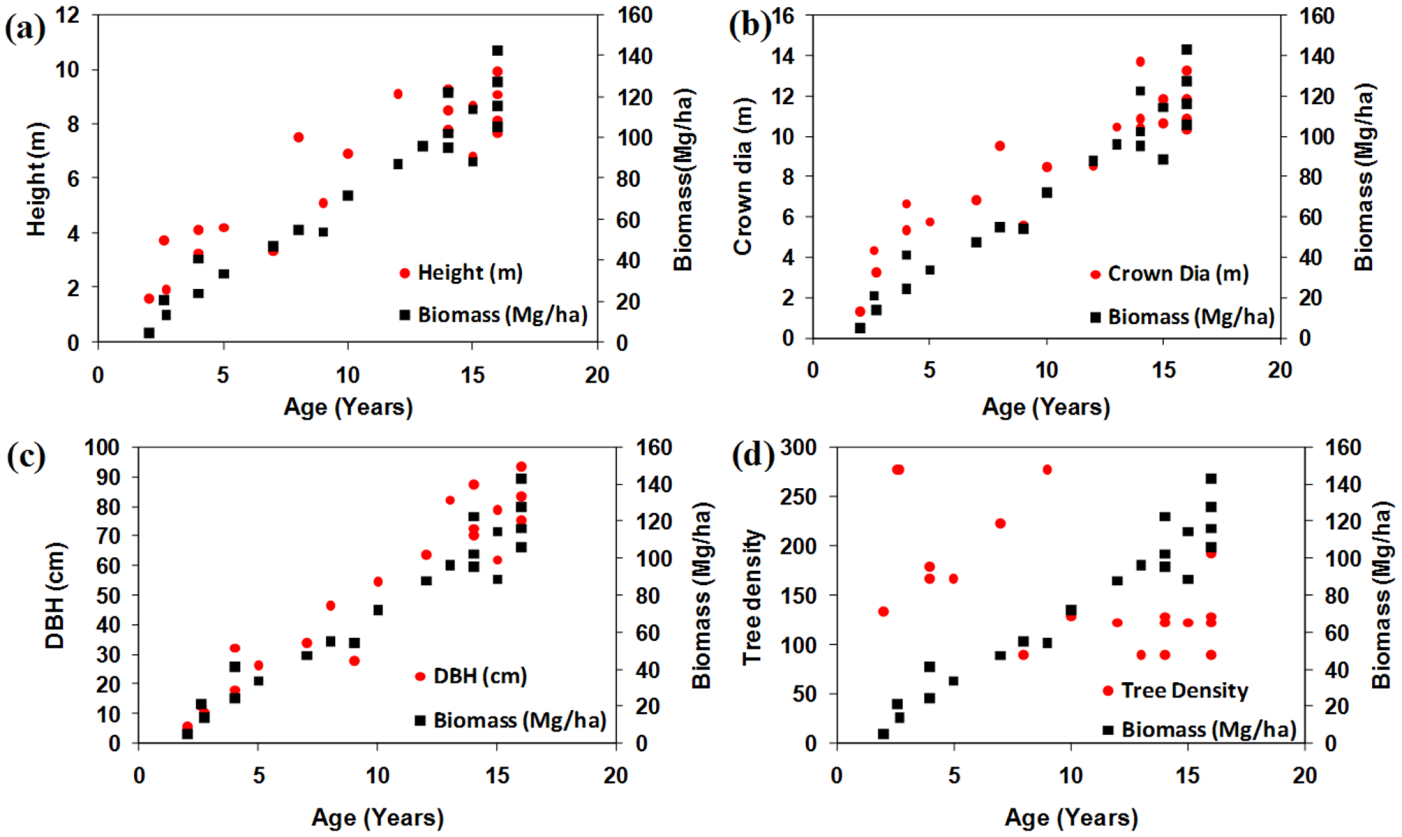
Relationship between age *vs* biomass with other biophysical parameters of cashew plants (a) height (b) crown diameter (c) DBH and (d) tree density respectively.

**Figure 5:**
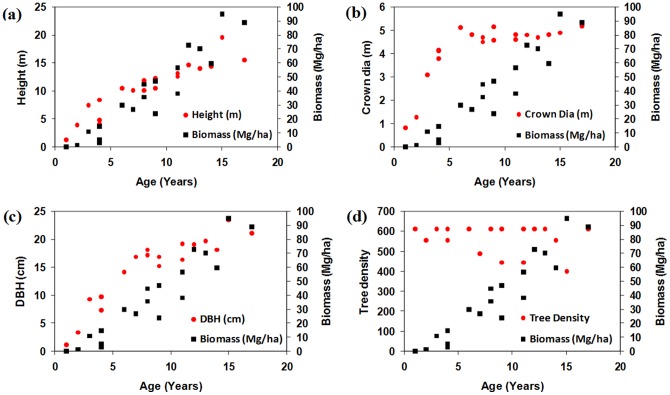
Relationship between age *vs* biomass with other biophysical parameters of rubber plants (a) height (b) crown diameter (c) DBH and (d) tree density respectively.

Statistical analyses were conducted to compare biophysical parameters of cashew and rubber plants. Figures 4 (a, b, c, d) and 5 (a, b, c, d) show relationships for height, crown diameter, diameter at breast height (DBH), and tree density with respect to age *vs.* biomass of cashew and rubber plants. Biophysical parameters of cashew plants showed positive relationship with plant age and biomass (Figure 4a, b, c), with the exception of tree density (Figure 4d). Biophysical parameters of rubber plants also showed positive relationship with plant growth and biomass (Figure 5a, c), except for crown diameter and tree density (Figure 5b, d). Cashew plants had greater biomass, DBH, and crown diameter compared with rubber plants at their mature stage. Tree density is a conditional parameter and depends on the species of the seedlings used. Variability in tree density in cashew plants arises because diseases and insects affect or kill trees that do not get enough light due to shade from neighbouring trees. However, in the case of rubber plants, tree density as a function of age is relatively stable. Rubber plants reach maturity after 4 years of growth and provide commercial yield. Field data also show that rubber plants do not show significant increases in crown diameter after approximately 6 years of growth (Figure 5d), whereas cashew plants show regular increases in crown diameter.

**Figure 6:**
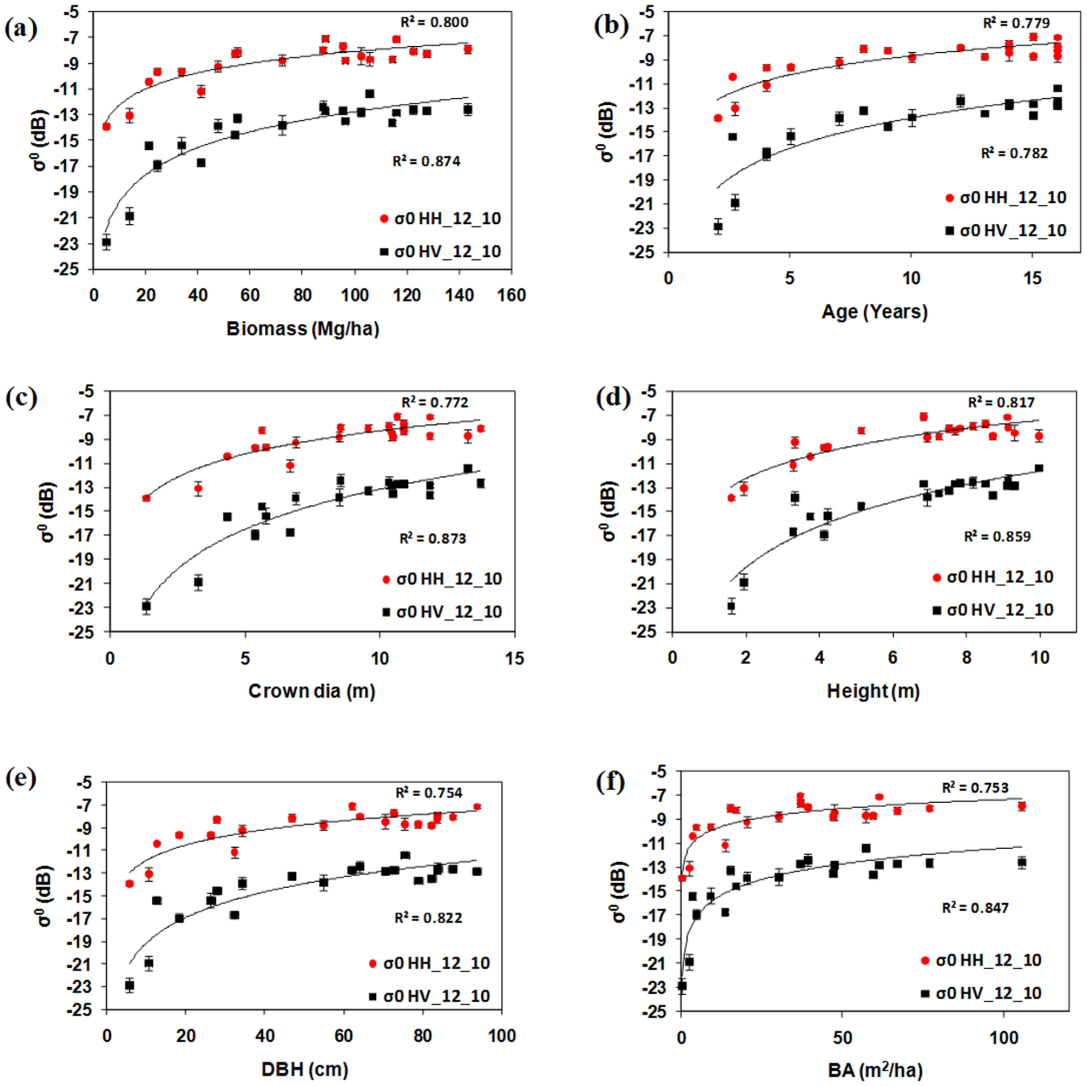
Relationship between PALSAR σ^0^ and biophysical parameters of cashew plants (a) biomass (b) age (c) crown diameter (d) height (e) DBH and (f) basal area respectively.

**Figure 7:**
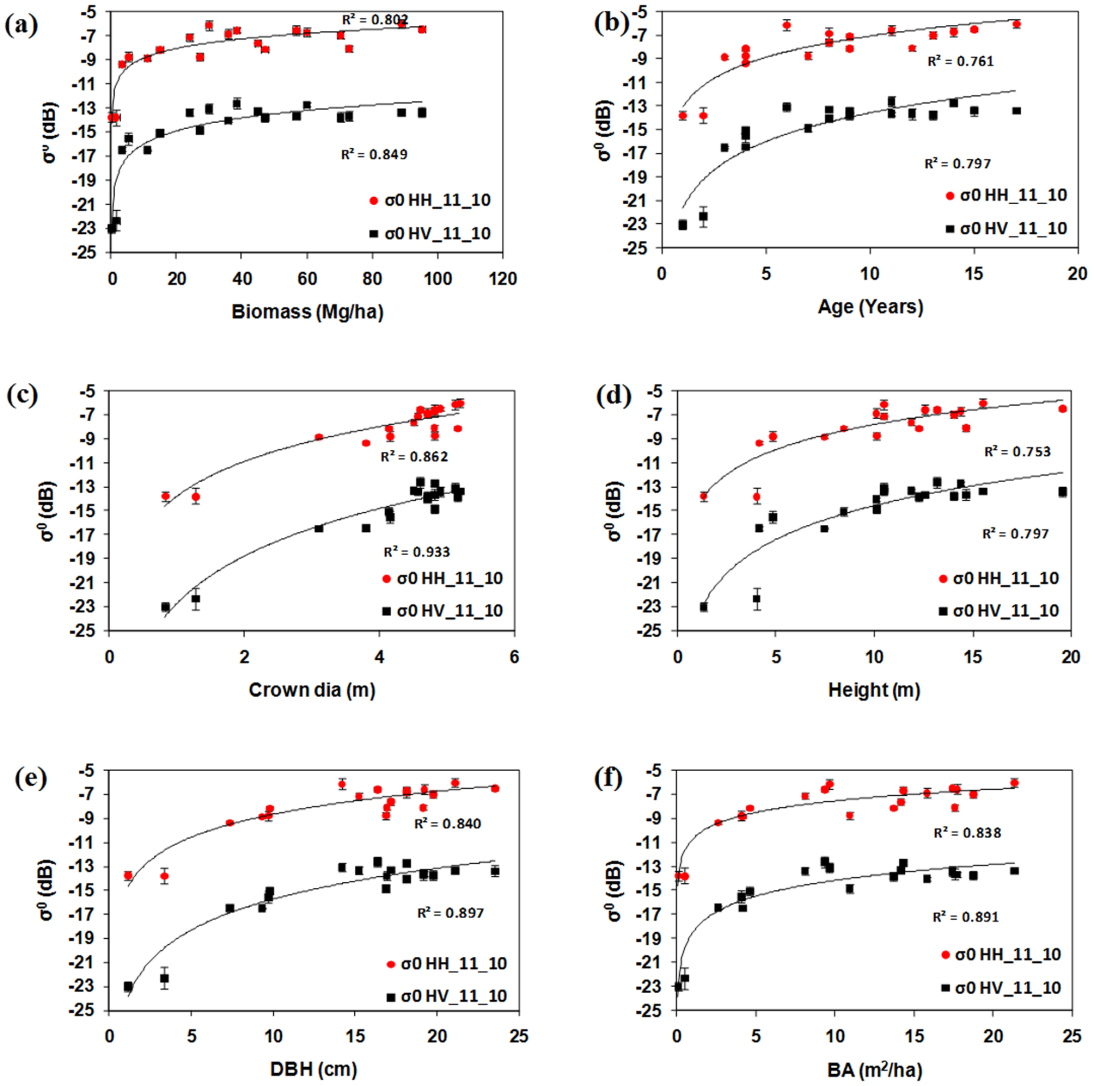
Relationship between PALSAR σ^0^ and biophysical parameters of rubber plants (a) biomass (b) age (c) crown diameter (d) height (e) DBH and (f) basal area respectively.

PALSAR data with HH and HV polarisation were analysed for their relationships with the cashew and rubber plant biophysical parameters. High penetration of L-band SAR showed a positive relationship with cashew and rubber plant biophysical parameters. Figures 6 (a, b, c, d, e, f) and 7 (a, b, c, d, e, f) show relationships between PALSAR backscattering and ground-based measurements of biophysical parameters of cashew and rubber plants. Although both HH and HV polarisation showed positive relationships with field-based data, the sensitivity of σ^0^ HV was higher than that of σ^0^ HH in both plantation types because of an increase in the volume of scattering with the growth of cashew and rubber plants. Low backscattering and high variation were seen at juvenile stages in cashew and rubber plants, mainly due to high growth rates during juvenile stages. After a certain amount of growth (4–5 years), cashew and rubber plants reached maturity and showed high backscattering. Because of fast growth during juvenile stages, the gaps between plants decreased considerably within 3 to 4 years after planting. During juvenile stages, plants had smaller leaves and fewer branches that caused less scattering, resulting in low σ^0^ HV. The gaps between canopies as growth proceeded gradually decreased, making the surface less visible. High backscattering and low variation were observed with mature cashew and rubber plants because of their homogeneous canopies. This increase in σ^0^ was mainly caused by growth in the plants' biophysical parameters.

Backscattering coefficient of HV polarization showed saturation around 100 Mg/ha biomass for cashew and 50 Mg/ha for rubber plants. Saturation of the PALSAR signal occurred after 13 years of growth in cashew plants. However, in rubber plants, saturation at 50 Mg/ha was because of saturation in crown cover. As in rubber plants, there was no significant increase in crown diameter after 6 years of growth. The rubber plant has a simple and symmetrical canopy, with homogeneous crown cover that becomes stable after 6 years of growth, whereas cashew plants show branching from the ground level, with a large crown diameter that increases with age and overlaps with adjacent trees. Therefore, cashew plants cause higher scattering volume in mature stages than rubber plants do. In this study, we observed that growth in the crown size of rubber and cashew plants was the main factor contributing to the volume of scattering [51]; [50]. We observed high R^2^ values of σ^0^ HH and HV with biophysical parameters of cashew and rubber plants. However, σ^0^ HV showed stronger correlations than σ^0^ HH with biophysical parameters in both plants previous studies also noticed strong correlation between HV polarization and plants biophysical parameters [50], [51].

### c. MLR model generation and application in a natural forest area

**Figure 8:**
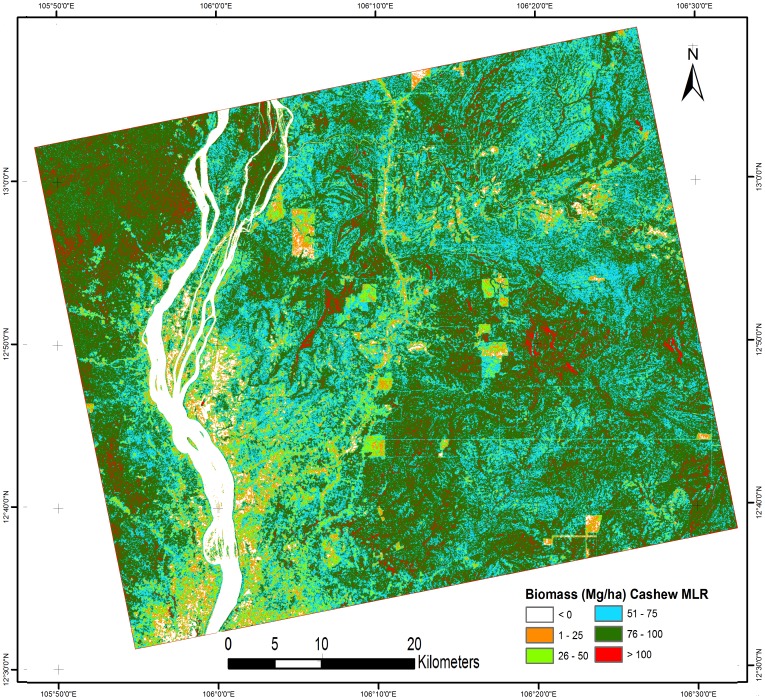
Natural forests biomass based on (cashew biomass MLR model 1).

**Figure 9:**
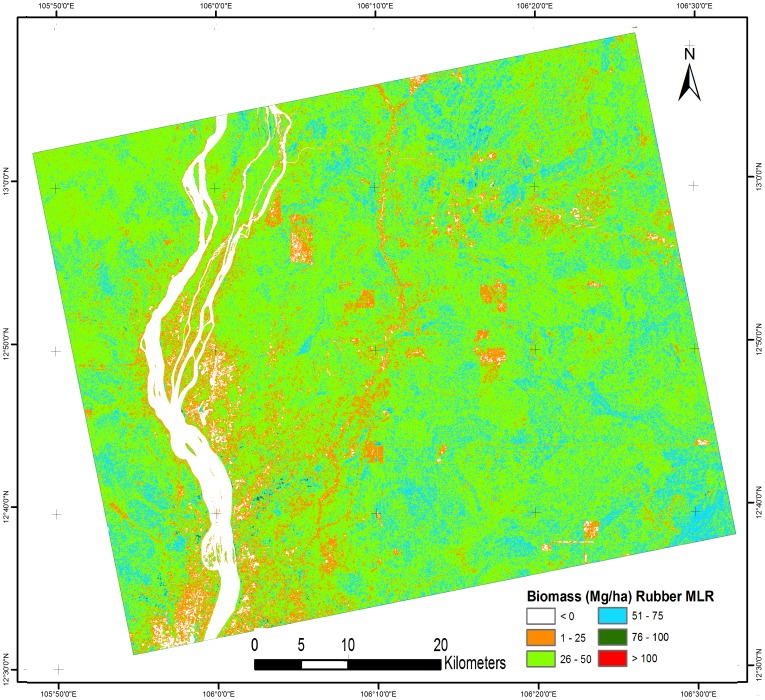
Natural forests biomass based on (rubber biomass MLR model 2).

**Figure 10:**
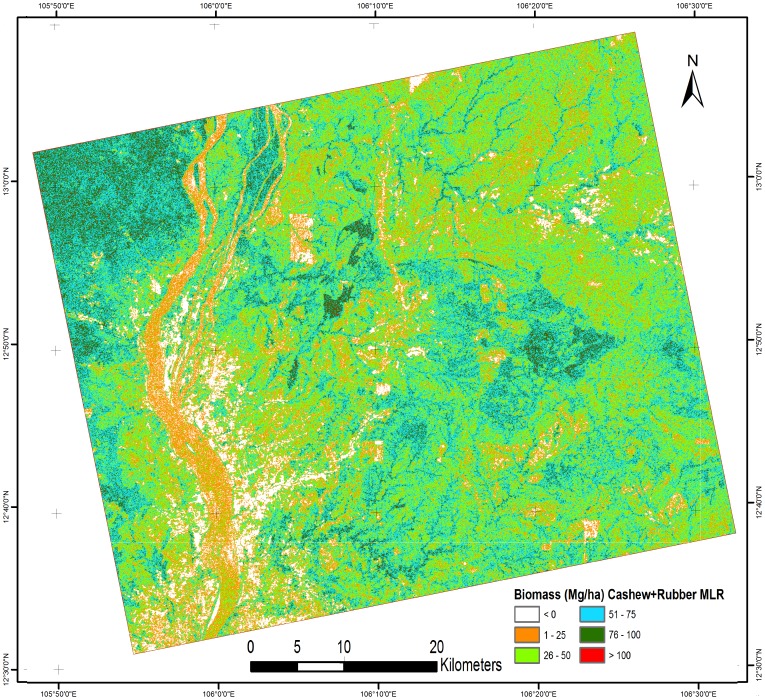
Natural forests biomass based on (cashew + rubber biomass MLR model 3).

Based on the above observations, three MLR models were created: Model 1 (cashew plant-based biomass), Model 2 (rubber plant-based biomass), and Model 3 (mixture of cashew and rubber plant-based biomass). Table 4 shows all three models with their R^2^ value. Model 1 had a high R^2^ value compared with models 2 and 3. These models were applied to PALSAR data from a natural forest area to predict tree biomass. The results of the AGB estimation for the natural forest are shown in Figures 8, 9 and 10 using models 1, 2, and 3, respectively. Table 5 shows the biomass distribution of the PALSAR-derived biomass map. From figure 8, it can be seen that throughout the deciduous forest, the amount of AGB was generally 51–100 Mg/ha. In some areas near a river, the AGB value was very low (1–25 Mg/ha) because most of the area was covered by agricultural land, where rice cultivation is common in the sampling season. In figure 8, the evergreen forest also shows an AGB value around 100 Mg/ha due to saturation of the PALSAR signal. Therefore, the application of model 1 in low-biomass regions, such as deciduous forests, is useful, but in high-biomass regions, such as evergreen forests, it shows saturation of the PALSAR signal. Figure 9 shows most of the deciduous forest area, where the amount of biomass varies from 26 to 50 Mg/ha. Even high-biomass regions such as evergreen forests show low-biomass values (50–75 Mg/ha). Thus, biomass prediction using model 2, which is based on the rubber plant, is not suitable. Figure 10 shows that in most of the deciduous area, the amount of biomass varies from 26 to 75 Mg/ha. The biomass prediction from model 3, which is based on both rubber and cashew plants, also resulted in underestimation and is not suitable for biomass prediction. Therefore, based on the regression results, we concluded that the methodological approach adopted in this study could be applied to estimate biomass in low-biomass regions such as deciduous forests. The results obtained from MLR model 1, which used cashew plant-based information, is the most promising among the models examined.

**Table 4:** MLR model based on cashew and rubber plants biomass.

No.	Plantation type	PALSAR Polarization	R^2^	R^2^ Adj	Regression Model No.
1	Cashew	HH, HV	0.63	0.59	Y(biomass) = 242.9+3.1×σ^0^HH+9.7×σ^0^HV
2	Rubber	HH, HV	0.43	0.36	Y(biomass) = 108.6+9.1×σ^0^HH−0.25×σ^0^HV
3	Cashew + Rubber (Mixed)	HH, HV	0.54	0.52	Y(biomass) = 206.1−12.7×σ^0^HH+17.6×σ^0^HV

**Table 5:** Percentage of biomass distribution for different biomass map.

No.	Biomass class (Mg/ha)	Cashew based MLR (% area) (Figure 8)	Rubber based MLR (% area) (Figure 9)	Cashew + Rubber based MLR (% area) (Figure 10)	Cashew – Rubber difference biomass (% area) (Figure 11)
1	0	5.54%	5.55%	6.67%	8.12%
2	1–25	3.92%	10.19%	16.66%	21.29%
3	26–50	8.65%	65.87%	37.91%	61.01%
4	51–75	28.46%	18.12%	30.16%	9.45%
5	76–100	49.38%	0.22%	8.01%	0.10%
6	>100	4.04%	0.04%	0.59%	0.03%
7	Total	100%	100%	100%	100%

**Figure 11:**
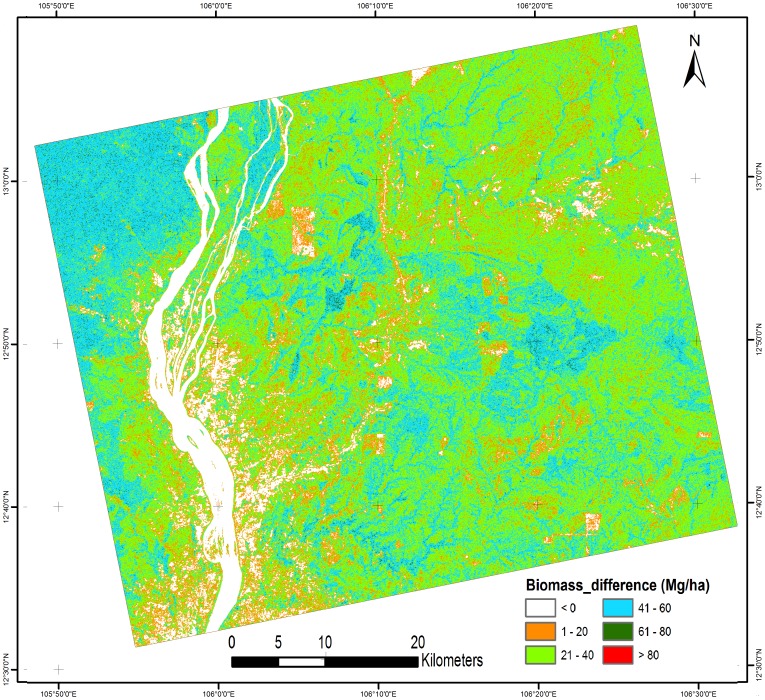
Biomass difference map (MLR model 1 – MLR model 2).

Figure 11 shows a biomass difference map comparing models 1 and 2. This map shows that the biomass in most of the area differed by 26–50 Mg/ha. We have observed that cashew plants show similar biomass patterns to deciduous forests in Cambodia. This model can be used to obtain a general idea of the biomass distribution. The plantation-based natural forest biomass estimation cannot provide accurate values in high-biomass regions because of saturation of the PALSAR signal. However, this technique is cost and time effective because the collection of forest inventory parameters in plantation areas is more convenient and has lower uncertainty compared with natural forests (Table 1). To overcome the saturation problem in high-biomass regions (100 Mg/ha), we need to examine P-band SAR or DESDynl satellites in the future [74]

### d. Validation

**Figure 12:**
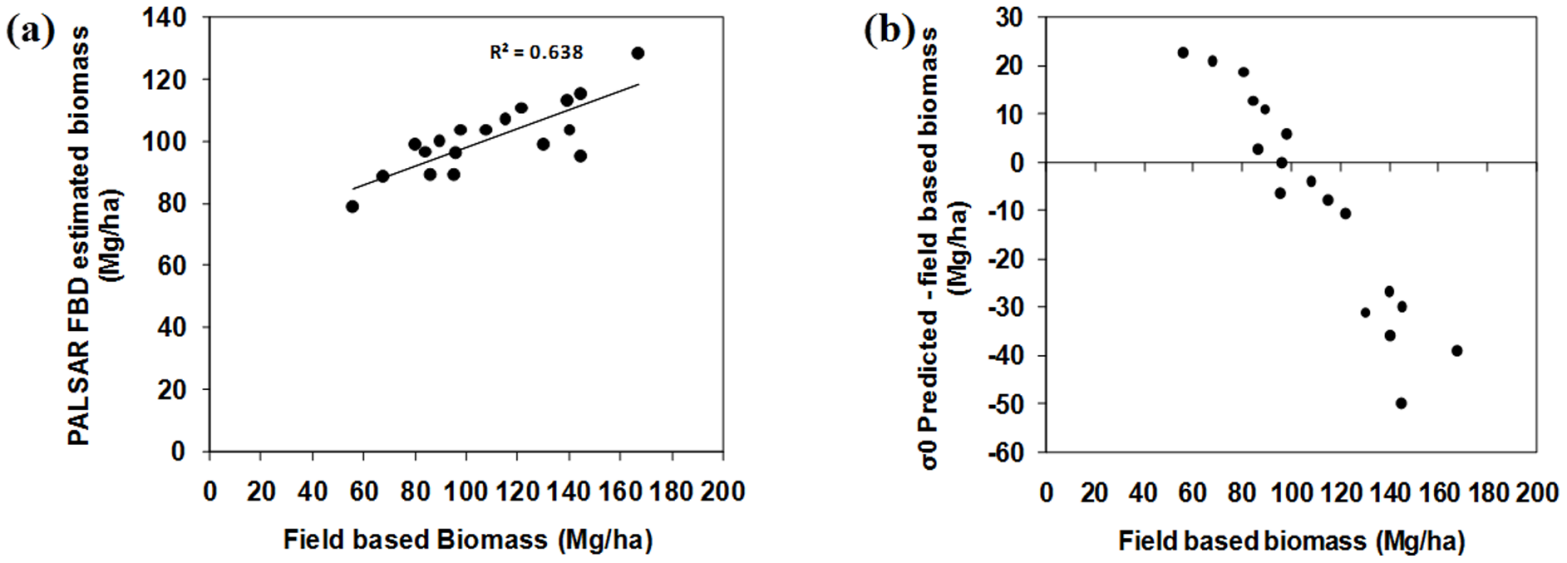
Validation results of biomass map (a) Relationship between PALSAR predicted natural forests biomass plotted against field measured biomass of natural forests (b) the error in PALSAR predicted natural forests biomass plotted against field measured biomass of natural forests.

Figure 12 (a, b) shows validation results for model 1-predicted (Figure 8) PALSAR-derived biomass with field measured biomass in natural forests. The sensitivity of PALSAR predicted AGB decreases as biomass increase because of saturation of the PALSAR signal. There was a significant correlation between model estimates and field measured biomass, R^2^ = 0.64. The overall root mean square error (RMSE) for the validation result was 23.2 Mg/ha. High variation in the error was present in high-biomass regions, i.e. where the biomass was greater than 100 Mg/ha (Figure 12b). Most of the high-biomass region showed underestimation by model 1.

## Conclusions

The relationships between biophysical parameters and PALSAR σ^0^ was investigated in cashew and rubber plantation areas. σ^0^ HV showed a higher positive correlation with field-based biomass data than did σ^0^ HH. Cashew plants showed saturation at 100 Mg/ha, whereas rubber plants showed saturation at 50 Mg/ha. In this study, we found that crown diameter was the main contributing factor to the volume of scattering. This study demonstrates that PALSAR data can be used to differentiate canopy structure. The development of plantation-based biomass-estimation models provides a new method to derive biomass in a cost-effective and timely way. The validation results showed a strong correlation between model estimations and field-based measurements (R^2^ = 0.64) with RMSE  = 23.2 Mg/ha in deciduous forests in Cambodia. We can use the generated biomass map to obtain a general idea of the biomass distribution because in high-biomass regions, this model becomes saturated. The advantage of this method is that is minimises cost, time, and labour during the collection of forest inventory data. The collection of inventory parameters is very easy and convenient, and it does not disturb the local ecosystem in plantation areas compared with natural forest areas (based on field experience). Further investigation into the use of full polarimetric PALSAR data with Cloude-Potteir, Freeman-Durden and Yamaguchi 4 component based decomposition parameters could help us to quantify and understand the scattering mechanisms from plantations and natural forests and their roles in forest biomass estimation.
